# Overexpression of *ATP1B1* predicts an adverse prognosis in cytogenetically normal acute myeloid leukemia

**DOI:** 10.18632/oncotarget.6226

**Published:** 2015-10-25

**Authors:** Jin-long Shi, Lin Fu, Qing Ang, Guo-jing Wang, Jun Zhu, Wei-dong Wang

**Affiliations:** ^1^ Medical Engineering Support Center, Chinese PLA General Hospital, Beijing, China; ^2^ Department of Hematology and Lymphoma Research Center, Peking University, Third Hospital, Beijing, China

**Keywords:** ATP1B1, expression, prognosis, CN-AML

## Abstract

*ATP1B1* encodes the Na,K-ATPase β subunit, a key regulator of the Na^+^ and K^+^ electrochemical gradients across the plasma membrane and an essential regulator of cellular activity. We used several microarray datasets to test the prognostic efficacy of *ATP1B1* expression in cytogenetically normal acute myeloid leukemia (CN-AML). Within the primary cohort (*n* = 157), high *ATP1B1* expression (*ATP1B1*^high^) was associated with shorter overall survival (OS) and event-free survival (EFS) (*P* = 0.0068, *P* = 0.0039, respectively). Similar results were also obtained in the European Leukemia Net (ELN) Intermediate-I genetic category (OS: *P* = 0.0035, EFS: *P* = 0.0007). Multivariable analyses confirmed *ATP1B1*^high^ is an independent predictor of shorter OS (*P* = 0.042) and EFS (*P* = 0.035). Analysis of another CN-AML cohort confirmed that *ATP1B1*^high^ is associated with shorter OS (*P* = 0.0046, *n* = 162). In addition, up-regulation of oncogenes/onco-microRNAs such as *MYCN*, *CCND2*, *CDK6*, *KIT* and *miR-155*, among others, was associated with *ATP1B1*^high^, which may be indicative of *ATP1B1's* leukemogenicity. Our results may improve risk stratification and indicate new therapeutic targets for CN-AML.

## INTRODUCTION

Cytogenetically normal acute myeloid leukemia (CN-AML) accounts for 40-50% of all AML [1] and shows significantly heterogeneous outcomes [2]. There are no microscopically detectable chromosome abnormalities in the leukemic blasts of CN-AML patients, but mutations, epigenetic changes and dysregulated expression signatures have been all found and used as biomarkers for prognostic evaluation and risk classification [3]. These include mutations of *NPM1* [4], *CEBPA* [5] and *FLT3*-*ITD* [6], as well as *WT1* [7], *DNMT3A* [8] and *TET2* [9], which are associated with an unfavorable prognosis. Other genes, microRNA and lncRNA [10] found to be associated with prognostic outcomes include *BAALC, ERG* [11], *WT1* [12], *DNMT3B* [13], *TCF4* [14], *ITPR2* [15], *MAPKBP1* [16], *miR*-*155* [17], *CXXC5* [18], *let-7a-2* and *miR-188* [19]. These biomarkers are useful indicators of the degree of malignancy in leukemia, and help focus targeted therapies. However, they do not clarify the treatment intensity necessary to optimize outcome, which is vital for the future lives of CN-AML patients. Consequently, identification of new biomarkers remains an urgent clinical need. In addition, considering that much about leukemogenic mechanisms remains unknown, new biomarkers that shed light on the underlying molecular events may increase our understanding of myeloid leukemogenesis.

Systematic screening for potential biomarkers has been carried out using several bioinformatics approaches with multiple *GEO* microarray datasets, and gene signatures that showed both aberrant expression and significant prognostic value were identified (See Figure S1). We previously showed that high expression of *ITPR2*, which encodes a key regulator of transmembrane calcium ion (Ca^2+^) transport, was predictive of an adverse outcome for CN-AML patients [15], which stimulated our interest in regulators of metal ion transport.

*ATP1B1* encodes Na,K-ATPase β subunit, an integral membrane protein essential for establishing and maintaining the Na^+^ and K^+^ electrochemical gradients across the plasma membrane. In addition, it was recently reported that targeting of Na,K-ATPase β subunit could induce apoptosis and cell cycle arrest [20], and that impairment of the Na,K-ATPase β subunit increased the incidence of apoptosis among leukemia cells [21, 22]. These results suggest that *ATP1B1* expression may be a useful indicator of prognosis in CN-AML patients.

## RESULTS

### Overexpression of *ATP1B1* in CN-AML

**Figure 1:**
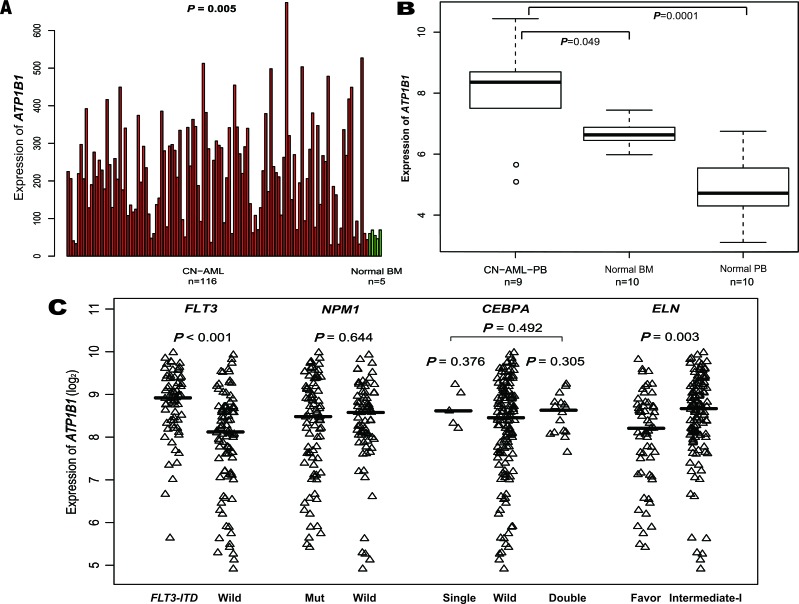
Differential expression of *ATP1B1* **(A)**. CN-AML and normal BM. **(B)**. CN-AML and normal BM and normal PB. **(C).**. *FLT3*-*ITD*, *NPM1*, mutant and wild-type *CEBPA*, ELN Favorable and Intermediate-I.

Expression of *ATP1B1* was significantly higher in bone marrow (BM) from the primary cohort of 157 CN-AML patients than in normal BM (*p* < 0.001, Figure 1A, 116 CN-AML *vs*. 5 normal BM, GEO No: *GSE1159*), and similar results were obtained using other microarray data (Figure 1B, 9 CN-AML *vs*. 10 normal BM *vs*. 10 normal peripheral blood (PB); the 9 CN-AML consisted of 2 BM and 7 PB samples, GEO No: *GSE9476*). After dividing the 157 CN-AML patients into subgroups based the mutation status of *FLT3*, *NPM1* or *CEBPA* (GEO No: *GSE6891*) (Figure 1C), *ATP1B1* showed significantly higher expression in samples with *FLT3-ITD* (*n* = 66) than without *FLT3-ITD* (*n* = 91) (*p* < 0.001). By contrast, there was no significant difference in *ATP1B1* expression between *NPM1*-mutated (*n* = 82) and wild-type samples (*n* = 75) (*p* = 0.644, Figure 1C), or between single *CEBPA*-mutated (*n* = 5), double *CEBPA*-mutated (*n* = 16) and wild-type samples (*n* = 133) (*p* = 0.376, *p* = 0.305 and *p* = 0.492, respectively, Figure 1C). Finally, significantly higher *ATP1B1* expression was detected in the European Leukemia Net (ELN) intermediate-I category than in the ELN-favorable category (*p* = 0.003, Figure 1C).

### Pretreatment clinical and molecular characteristics associated with *ATP1B1*^high^

**Figure 2:**
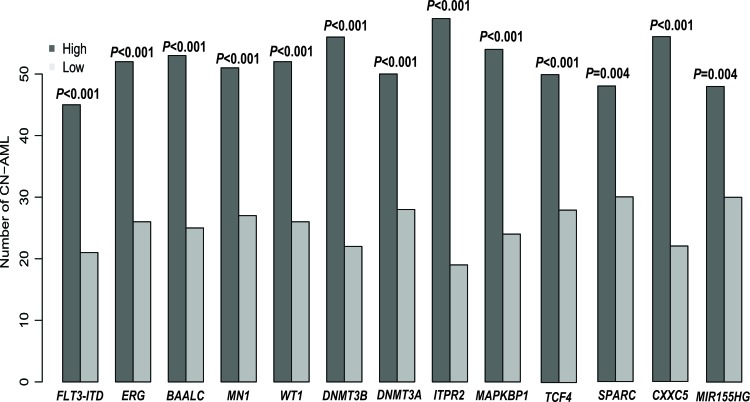
Associations between *ATP1B1* expression and known prognostic biomarkers

Among the primary cohort, *ATP1B1*^high^ expressers included more patients with FAB M1 and M2 (*p* = 0.02, *p* = 0.0015, respectively) and fewer with FAB M4 and M5 (*P* = 0.19, *p* = 0.0008, respectively). In the ELN genetic categories, more *ATP1B1*^high^ expressers were within intermediate-I (*P* = 0.002), while fewer were within favorable genetic categories. More *ATP1B1*^high^ expressers also carried *FLT3-ITD*, and several known unfavorably prognostic biomarkers were up-regulated in CN-AML (*ERG*, *BAALC*, *MN1*, *WT1*, *DNMT3B*, *TCF4*, *ITPR2*, *DNMT3A*, *SPARC*, *CXXC5*, *MAPKBP1* and *MIR155HG*) (See Table 1 and Figure 2).

**Table 1:** Clinical characteristics of the 157 CN-AML patients in the primary cohort segregated based on the *ATP1B1* expression levels

Variable	*ATP1B1*^high^, *n* = 78	*ATP1B1*^low^, *n* = 79	*P*
Median age. y (range)	49.5 (16-77)	50 (18-77)	0.38
Female sex, no.(%)	35 (44.9)	38 (48.1)	0.75
FAB subtype, no.			
M0	2	1	0.62
M1	29	16	0.02
M2	24	8	0.0015
M3	1	0	0.5
M4	9	15	0.19
M5	10	29	0.0008
M6	0	1	1
Other	3	9	0.13
*FLT3*-*ITD*, presented, no.	45	21	<0.0001
*NPM1*, mutated, no.	39	43	0.63
*CEBPA*, single mutated, no.	3	2	0.68
*CEBPA*, double mutated, no.	9	7	0.5
*FLT3*-*TKD*, presented, no.	7	13	0.23
*N*-*RAS*, mutated, no.	4	9	0.25
*K*-*RAS*, mutated, no.	0	1	1
*IDH1*, mutated, no.	10	9	0.8
*IDH2*, mutated, no.	8	5	0.4
ELN genetic group, no.			
Favorable	24	35	0.1
Intermediate-I	69	53	0.002
High *ERG*, no.	52	26	<0.0001
High *BAALC*, no.	53	25	<0.0001
High *LEF1*, no.	39	39	1
High *MN1*, no.	51	27	0.0001
High *WT1*, no.	52	26	<0.0001
High *DNMT3B*, no.	56	22	<0.0001
High *TCF4*, no.	50	28	0.0004
High *ITPR2*, no.	59	19	<0.0001
High *MIR155HG*, no.	48	30	0.004
High *MAPKBP1*, no.	54	24	<0.0001
High *DNMT3A*, no.	50	28	0.0004
High *SPARC*, no.	48	30	0.004
High *CXXC5*, no.	56	22	<0.0001

Abbreviations: FAB, French-American-British classification; ITD, internal tandem duplication; TKD, tyrosine kinase domain; ELN, European Leukemia Net.

High *ERG*, *BAALC*, *LEF1*, *MN1*, *WT1*, *DNMT3B*, *TCF4, ITPR2, DNMT3A, MIR155HG, MAPKBP1, SPARC* and *CXXC5* expression were defined as an expression level above the median of all samples, respectively.

### Prognostic value of *ATP1B1* expression

**Figure 3:**
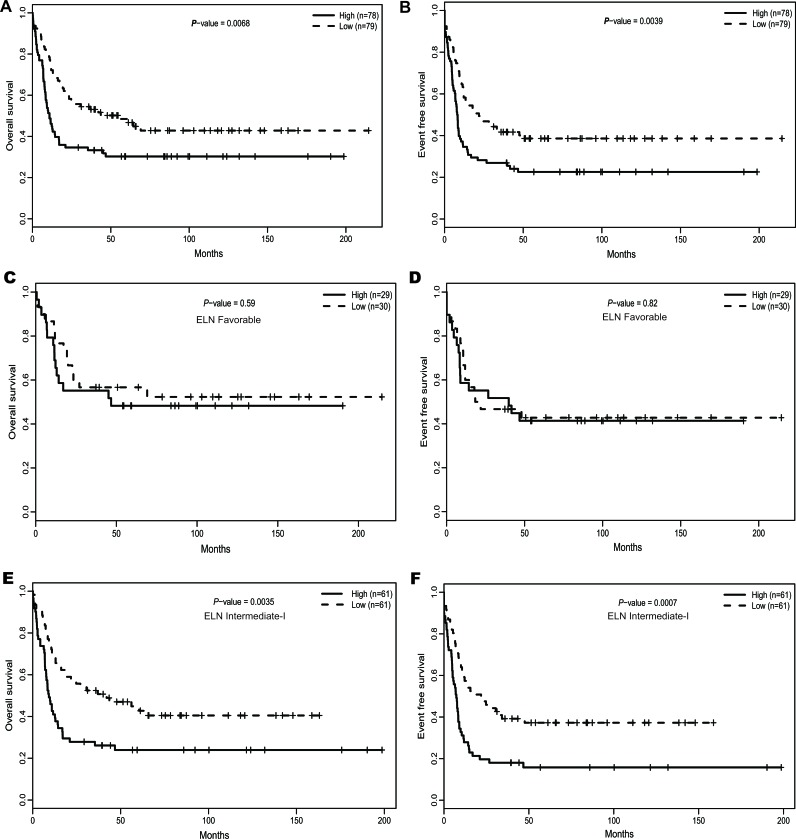
The prognostic value of *ATP1B1* expression **(A).** OS and **(B).** EFS among the 157 CN-AML patients in the primary cohort. **(C).** OS and **(D).** EFS in the ELN Favorable category. **(E).** OS and **(F).** EFS in the ELN Intermediate-I category.

After dividing the 157 CN-AML patients into two groups based on the median *ATP1B1* expression level, *ATP1B1*^high^ expressers showed significantly shorter overall survival (OS) (*P* = 0.0068, Figure 3A) and event-free survival (EFS) (*P* = 0.0039, Figure 3B). Further, *ATP1B1*^high^ expressers were respectively 1.56 times and 1.55 times as likely to die in the OS (*P* = 0.042) and EFS (*P* = 0.035) multivariable models (See Table 2) after adjustment of several known prognostic factors. We also analyzed the prognostic impact of *ATP1B1* expression within ELN genetic categories. In the favorable group (*n* = 59), no significant differences were detected for OS and EFS (See Figure 3C and 3D). In the intermediate-I group (*n* = 122), however, *ATP1B1*^high^ patients were associated with dramatically shorter OS (*P* = 0.0035, Figure 3E) and shorter EFS (*P* = 0.0007, Figure 3F). In addition, *ATP1B1*^high^ expressers were significantly associated with shorter estimated OS/EFS at 3 years, whether among the entire 157 CN-AML patient cohort or in the ELN intermediate-I category (See Table S1).

**Table 2:** Multivariable analysis with OS and EFS in the primary cohort of 157 CN-AML patients

Variable	OS, n=157		EFS, n=157	
	HR(95% CI)	P	HR(95% CI)	P
*ATP1B1* expression, high *VS* low	1.56(1.02-2.40)	0.042	1.55(1.03-2.33)	0.035
Age, per 10-y increase	1.15(0.99-1.35)	0.070	1.06(0.91-1.23)	0.434
Sex male *VS* female	0.79(0.52-1.19)	0.263	0.96(0.65-1.41)	0.827
*FLT3-ITD*, presented *VS* others	1.93(1.21-3.07)	0.006	1.74(1.12-2.72)	0.014
*NPM1,* mutated *VS* wild type	0.55(0.34-0.87)	0.010	0.57(0.37-0.88)	0.012
*CEBPA*, single mutated *VS* wild type	0.79(0.32-1.95)	0.607	0.92(0.37-2.25)	0.850
*CEBPA*, double mutated *VS* wild type	0.49(0.21-1.11)	0.088	0.51(0.24-1.07)	0.075

### Confirmation that *ATP1B1*^high^ is an unfavorable biomarker using an independent cohort

The prognostic value of *ATP1B1* expression was further confirmed in an independent cohort of 162 CN-AML patients (GEO no: *GSE12417*), (*P* = 0.005, See Figure S3). Using a cutoff of median expression, *ATP1B1*^high^ expressers belonged more to FAB M1 and less to FAB M5 than *ATP1B1*^low^ expressers (*P* = 0.005, *P* = 0.048, respectively). *ATP1B1*^high^ was also associated with higher expression of *ERG*, *BAALC*, *MN1*, *WT1*, *TCF4*, *SPARC*, *DNMT3B* and *ITPR2* (All *P* < 0.001, See Table S2 and Figure S2), as was the case with the primary cohort.

### Genome-wide gene expression profiles associated with *ATP1B1*^high^

**Figure 4:**
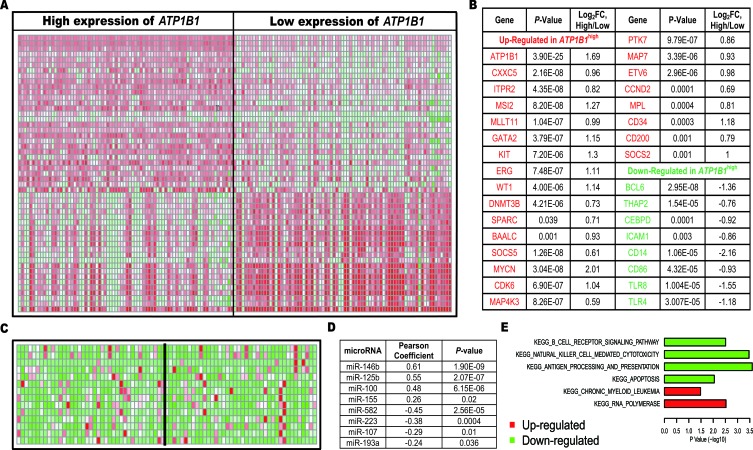
Genes/microRNAs/cell signaling pathways associated with high *ATP1B1* expression **(A)**. Gene expression heat map. **(B)**. List of genes. **(C)**. MicroRNA expression heat map. **(D)**. List of microRNAs. **(E)**. Pathways.

To investigate the mechanisms by which *ATP1B1* expression affects outcome in CN-AML, we first performed a genome-wide differential analysis after again subdividing the primary cohort of CN-AML patients based on the median *ATP1B1* expression. In the *ATP1B1*^high^ group, 608 genes were up-regulated and 975 were down-regulated as compared to *ATP1B1*^low^ (False Discovery Rate, FDR < 0.05; absolute fold change, FC ≥ 1.5, See Table S3). Notably, many genes known to be associated with worse outcomes in CN-AML/AML were up-regulated, including *WT1* [12], *ITPR2* [15], *MAPKBP1* [16], *BAALC*, *ERG* [11], *MN1* [23], *SPARC* [24], *DNMT3B* [13], *MSI2* [25], *GATA2* [26], *SOCS2 [27]*, *CXXC5* [18] and *MLLT11* [28]. The up-regulated genes also included well-known oncogenes and those previously found to be involved in leukemogenesis, including *MYCN*, *KIT* [29], *CCND2* and *CDK6*. The encoded proteins include a cyclin kinase, two mitogen-activated protein kinases (*MAP4K3*, *MAPK7*) and a tyrosine kinase (*PTK7*), which are involved in regulating gene transcription and cell proliferation and differentiation. Other up-regulated genes included *CD34*, which acts as a marker for hematopoietic progenitor cells, *MPL*, which initiates and maintains *RUNX1*-*ETO* AML [30], *CD200*, whose up-regulation can promote AML progression [31], *SOX4*, which is a key oncogenic target in C/EBPα mutant AML [32], *ETV6*, which often cooperates with other oncogenic signals to induce leukemia [33, 34], and *MAP7*, which is targeted by miR-16 and regulates cell proliferation and the cell cycle in several cancer cell lines [35]. The down-regulated genes included *BCL6*, *THAP2*, *ICAM1* and *CEBPD* as well as those encoding members of the toll-like receptor family (*TLR4* and *TLR8*), which can function in pathogen recognition and activation of innate immunity, and *CD14* and *CD86*, which can induce T-cell activation and related immune responses (Figure 4A and 4B).

We used pathway data provided by MSgiDB to evaluate cell signaling pathways associated with *ATP1B1*^high^. Pathway expression was estimated as the averaged expression of all involved genes. We found that important apoptotic and natural killer signaling pathways were significantly down-regulated, while RNA polymerase and CML pathways were significantly up-regulated (Figure 4E). These findings are consistent with the gene dysregulation summarized above, and suggest why *ATP1B1* was associated with unfavorable outcomes in CN-AML.

### Genome-wide miRNA profiles associated with *ATP1B1* expression

Screening for differences in microRNA expression using high-throughput sequencing data from 79 CN-AML patients provided by *TCGA*, we identified 50 differentially expressed microRNAs (FDR < 0.05), including 38 that correlated positively with *ATP1B1* expression and 12 that correlated negatively (See Table S4). Those positively correlated microRNAs included *miR-146b, miR-125b, miR-100* and *miR-155* (*P* = 0.02). MiR-146b binds to the 3′UTR of *SMAD4*, an important member of TGF-β signaling pathway [36]. *MiR-155* was previously confirmed to be an independent biomarker of a poorer prognosis in CN-AML [17]. Overexpression of *miR-125b* was shown to independently induce leukemia in a mouse model [37]. And *miR-100* blocks the terminal differentiation of myeloid cell by targeting *RBSP3* [38]. The most negatively correlated microRNA was *miR-582*, which reportedly inhibits bladder cancer progression [39]. Other negatively correlated microRNAs included *miR-107* and *miR-223. miR-107* targets *CEBPA,* which binds to the promoter of *miR-223*, ultimately inhibiting differentiation of granulocytic cells [40]. *MiR-193a* targets *c-KIT* and acts as a tumor suppressor in AML (See Figure 4C and 4D) [29].

### Genome-wide methylation profiles associated with *ATP1B1* expression

DNA methylation is an important epigenetic mechanism that regulates gene expression, and some methylation loci have been used to predict clinical outcomes in CN-AML [3]. Considering that expression of both *DNMT3A* and *DNMT3B* correlated positively with *ATP1B1* expression, we carried out a genome-wide differential analysis to identify differences in the methylation patterns between *ATP1B1*^high^ and *ATP1B1*^low^ expressers based on a *TCGA* AML cohort of 74 CN-AML patients. Surprisingly, comparison of the methylation levels at several different regions (*TSS200*, *TSS1500*, *Promoter*, *Body*, *3′UTR* and *5′UTR*, See Figure S4) revealed no significant differences in methylation associated with *ATP1B1* expression status.

## DISCUSSION

*ATP1B1* encodes an integral membrane ion pump responsible for maintenance of the Na^+^ and K^+^ electrochemical gradients across the plasma membrane, and contributes to the establishment of epithelial cell polarity [41] and cell adherence. In an earlier study we showed that strong expression of *ITPR2*, which encodes a key membrane Ca^2+^ transporter, is predictive of an unfavorable outcome in CN-AML [15]. The present study demonstrates that overexpression of *ATP1B1* may also predict a poor prognosis in CN-AML patients. Together, these studies suggest that metal ion (e.g., Ca^2+^, K^+^, Na^+^) transport status has an important impact on outcome in CN-AML.

Our first main finding is that *ATP1B1* is more strongly expressed in CN-AML than normal BM (Figure 1A and 1B), which suggests *ATP1B1* plays an active role during the pathogenesis of leukemia. The higher *ATP1B1* expression was easily detected using qPCR, which is readily applicable for clinical use. In addition, patients with higher *ATP1B1* expression were classified mainly into the M1 and M2 FAB subgroups, with fewer in the M5 subgroup, suggesting that *ATP1B1*^high^ expressers carry more immature cells, which likely indicates greater malignancy. *ATP1B1*^high^ is significantly associated with *FLT3*-*ITD* and shows higher expression in ELN Intermediate-I than in the Favorable category, which are consistent with *ATP1B1*'s prognostic role. However, *ATP1B1* shows only a trend toward lower expression in CN-AML patients with *NPM1* mutation, as well as a slight trend toward higher expression in patients with single or double *CEBPA* mutation, which is not consistent with the known prognostic role of *CEBPA* mutations. Perhaps there were too few samples with *CEBPA* mutations (5 with single and 16 with double mutations), and the discrepancy reflects an aberrant result that does not reflect the true efficacy of *CEBPA* mutation as prognostic indicator.

We also demonstrated that high *ATP1B1* expression was an unfavorable prognostic biomarker for CN-AML patients, based on clinical and molecular characteristics prior to treatment and OS/EFS across the entire cohort or within ELN categories. These results indicate that *ATP1B1* expression could be used for risk stratification, not only with respect to the entire CN-AML patient population, but also within ELN intermediated-I categories, which could facilitate design of more suitable therapies and has important clinical significance. The prognostic value of *ATP1B1* was confirmed in another cohort of 162 CN-AML patients. Moreover, to avoid possible distortion of the microarray data, high-throughput sequencing data from TCGA was used as a second independent validating dataset, which included 41 CN-AML patients treated with uniform chemotherapy (*P* = 0.039, See Figure S5). All of these results show that overexpression of *ATP1B1* is predictive of an adverse outcomes in CN-AML. Because there are no recurrent chromosomal alterations, CN-AML shows a degree of genetic uniformity, which facilitates identification of new biomarkers but limits the scope of their application. We therefore evaluated the prognostic value of *ATP1B1* in a cohort of 344 AML samples containing a variety of karyotypes. Notably, overexpression of *ATP1B1* was associated with a shorter OS and EFS (*P* = 0.027 and *P* = 0.0169, respectively. See Figure S6), indicating that overexpression of *ATP1B1* may be an unfavorable biomarker in both CN-AML and AML.

To investigate the potential mechanisms by which *ATP1B1* expression affects patient outcome, we carried out a multi-omics analysis. The first gene/microRNA expression profiles and methylation loci associated with *ATP1B1* expression were determined, including related cell signaling pathways. Many known oncogenes and unfavorable CN-AML biomarkers were found to be up-regulated, while various tumor suppressors and immune factors were down-regulated. In addition, important apoptotic and natural killer signaling pathways were all significantly down-regulated, while RNA polymerase and CML pathways were significantly up-regulated. These aberrant changes to the transcriptome likely contribute to the unfavorable outcomes for CN-AML.

MicroRNA-involved regulation and methylation are important epigenetic mechanisms, which play essential roles in many biologic processes, including tumorigenesis. In this study, several microRNAs known to be involved in tumorigenesis were found to be associated with *ATP1B1* expression, and may represent potentially useful therapeutic targets. No methylation patterns were associated with *ATP1B1* expression. Perhaps, demethylation agents would be of no use for patients strongly expressing *ATP1B1*.

In conclusion, our work demonstrates that overexpression of *ATP1B1* may be a useful unfavorable biomarker for evaluating CN-AML outcomes. Because of its higher expression in CN-AML patients than healthy individuals, *ATP1B1* may be easily detected using qPCR in clinical applications. Furthermore, our genome-wide analysis of aberrant gene/microRNA expression and cell signaling may lead to a better understanding of potential leukemogenic mechanisms, thereby aiding development of new therapeutic strategies for the treatment of CN-AML.

## MATERIALS AND METHODS

### Patient samples

This research was approved by the institutional review boards at Weill Cornell Medical College and Erasmus University Medical Center, and all patients provided written informed consent in accordance with the Declaration of Helsinki [47]. The study participants were 157 primarily untreated CN-AML patients (median age: 50 years, range: 16-77 years). Among them, 130 (83%) were aged < 60 years (younger patients) and 27 (17%) were ≥ 60 years (older patients). Patients were uniformly treated according to the study protocols of the Dutch-Belgian Hematology-Oncology Cooperative Group (HOVON, http://www.hovon.nl). BM aspirates and PB were collected at the time of diagnosis at the Erasmus University Medical Center (Rotterdam) between 1990 and 2008 [45]. The samples all contained 80%-100% blast cells after thawing [46]. To make a diagnosis of a normal karyotype, more than 20 metaphases from BM were examined using conventional cytogenetics. *NPM1*, *CEBPA*, *IDH1* and *IDH2* mutations; FLT3-internaltandem duplications (*FLT3-ITD*); and *N-RAS*, *K-RAS* and FLT3-tyrosine kinase domain mutations (*FLT3-TKD* [D835]) were all assessed. An independent validating cohort of 162 CN-AML patients all received uniform treatment (intensive double induction and consolidation chemotherapy) based on the multicenter AMLCG-1999 trial of the German AML Cooperative Group between 1999 and 2003 [48]. The AMLCG-1999 clinical trials were approved by the local institutional review boards, and all patients provided written informed consent in accordance with the Declaration of Helsinki.

### Microarray studies

All the microarray data used in our study were derived from the Gene Expression Omnibus (GEO) and were available for public downloading. Expression data for the 157 CN-AML patients in the primary cohort were detected using an Affymetrix HG-U133Plus 2.0 array [45], while the validating cohort of 162 CN-AML patients was evaluated using an Affymetrix HG-U133A array [49]. High-throughput sequencing data from The Cancer Genome Atlas (TCGA) were also used [50], including mRNA, microRNA and methylation data.

### Statistical analysis

Samples were divided into two groups (*ATP1B1*^high^, *n* = 78; *ATP1B1*^low^, *n* = 79) based on the median *ATP1B1* expression level. In addition, high and low classifications of *ERG*, *BAALC*, *WT1*, *LEF1*, *MN1*, *EVI1*, *DNMT3B*, *TCF4*, *ITPR2*, *MAPKBP1* and *MIR155HG* were determined according to the median expression of the corresponding genes. When comparing the *ATP1B1*^high^ and *ATP1B1*^low^ patient groups, Fisher's exact test and the Wilcoxon rank-sum test were used to compare categorical and continuous variables, respectively. Kaplan-Meier and log-rank test were used for survival analysis. Multivariable Cox proportional hazards models were used to study the time-to-event factors associated with survival endpoints. Student's *t*-test was used to identify *ATP1B1*-associated genes, pathways and methylation sites. Pearson correlation test was performed to detect correlated expression between *ATP1B1* and microRNA sequencing profiles. All analyses were performed using R 3.1.1.

## SUPPLEMENTARY MATERIAL FIGURES AND TABLES






