# Supplementary material for: Overexpression of ATP1B1 predicts an adverse prognosis in cytogenetically normal acute myeloid leukemia
Source: Oncotarget. 2015 Oct 25;7(3):2585–95. doi: 10.18632/oncotarget.6226 (PMC4823057; doi:10.18632/oncotarget.6226)
Supplement: Supplementary file 1 [file oncotarget-07-2585-s001.pdf]

Overexpression of *ATP1B1* predicts an adverse prognosis in cytogenetically normal acute myeloid leukemia

Supplementary Material

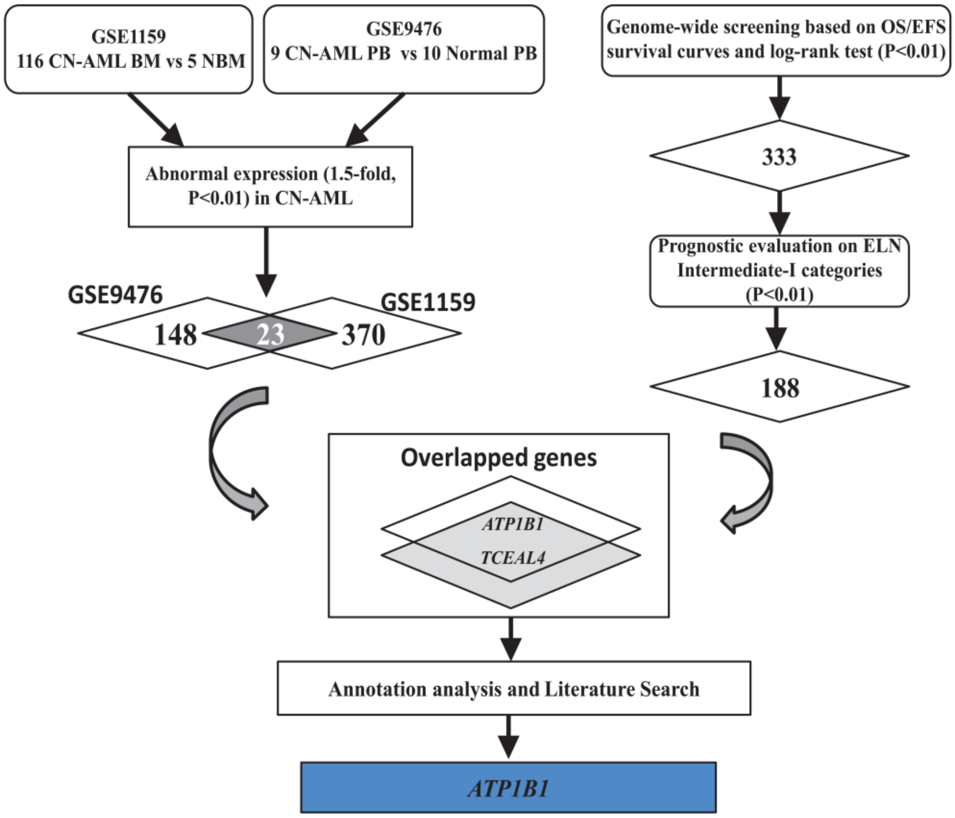

Figure S1. Flowchart of selecting *ATP1B1*.

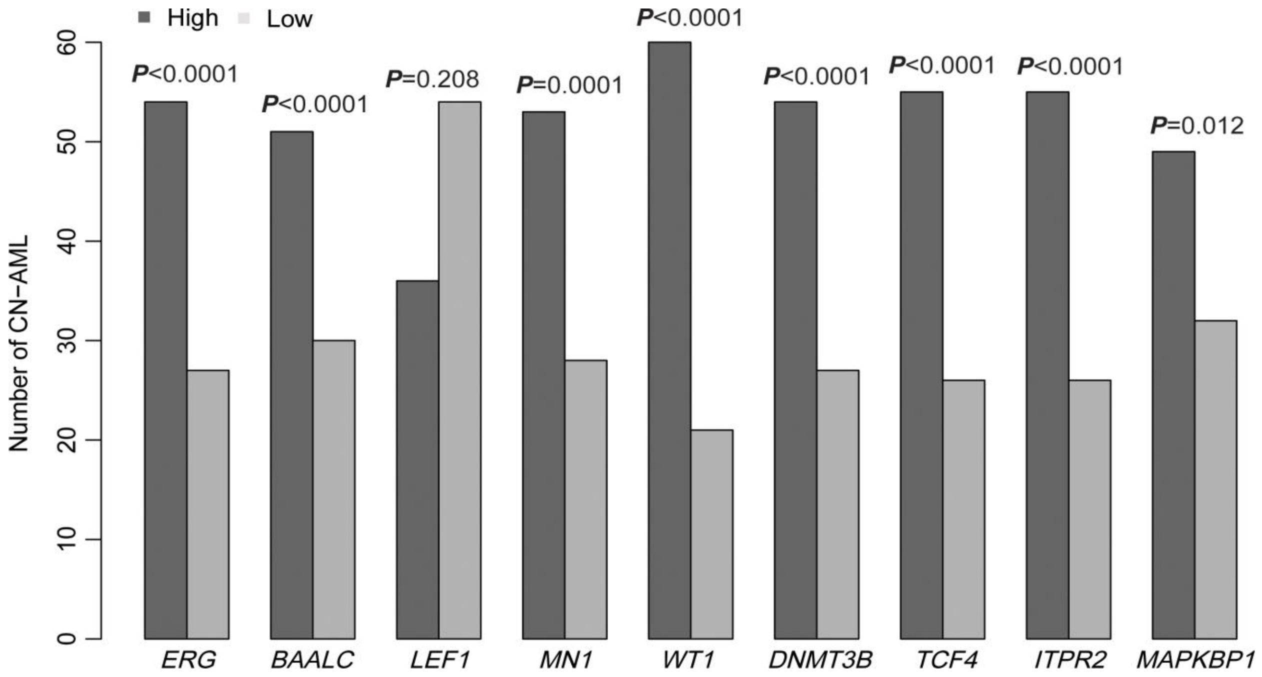

Figure S2. Association between *ATP1B1* expression and known prognostic biomarkers in the validating cohort (162 CN-AML).

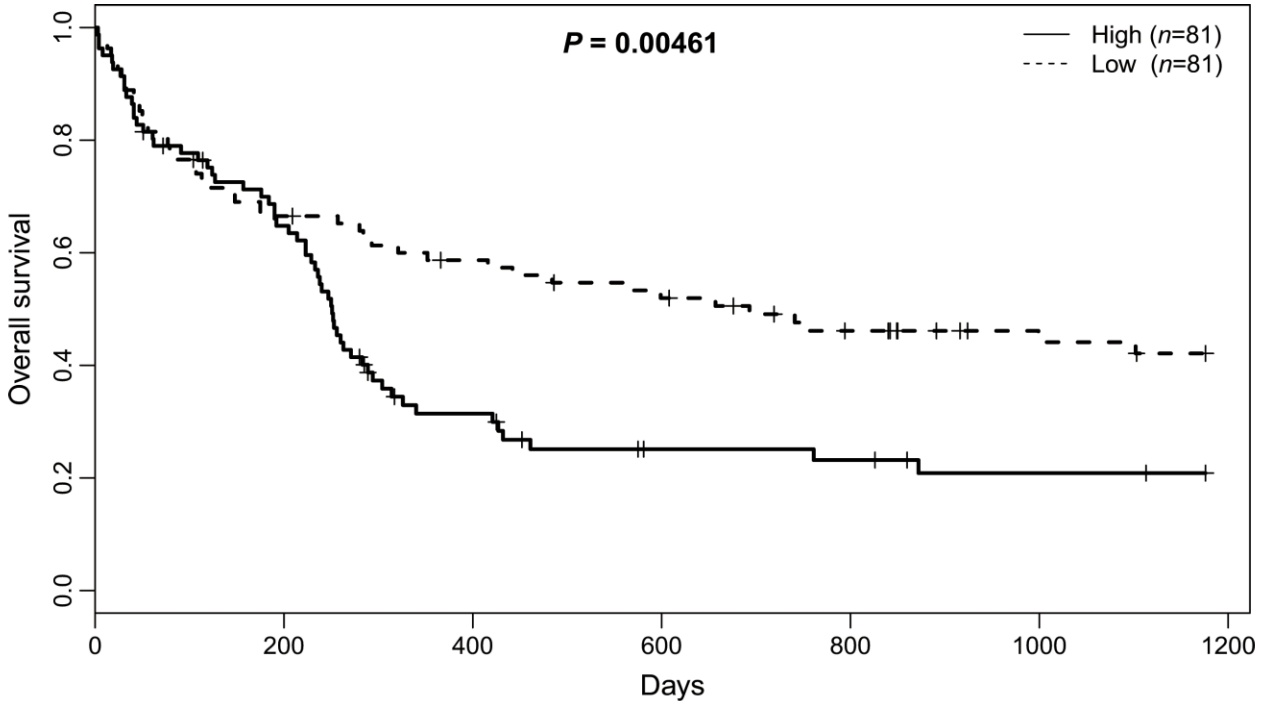

Figure S3. Overexpression of *ATP1B1* predicts shorter OS in the validating cohort of 162 CN-AML patients.

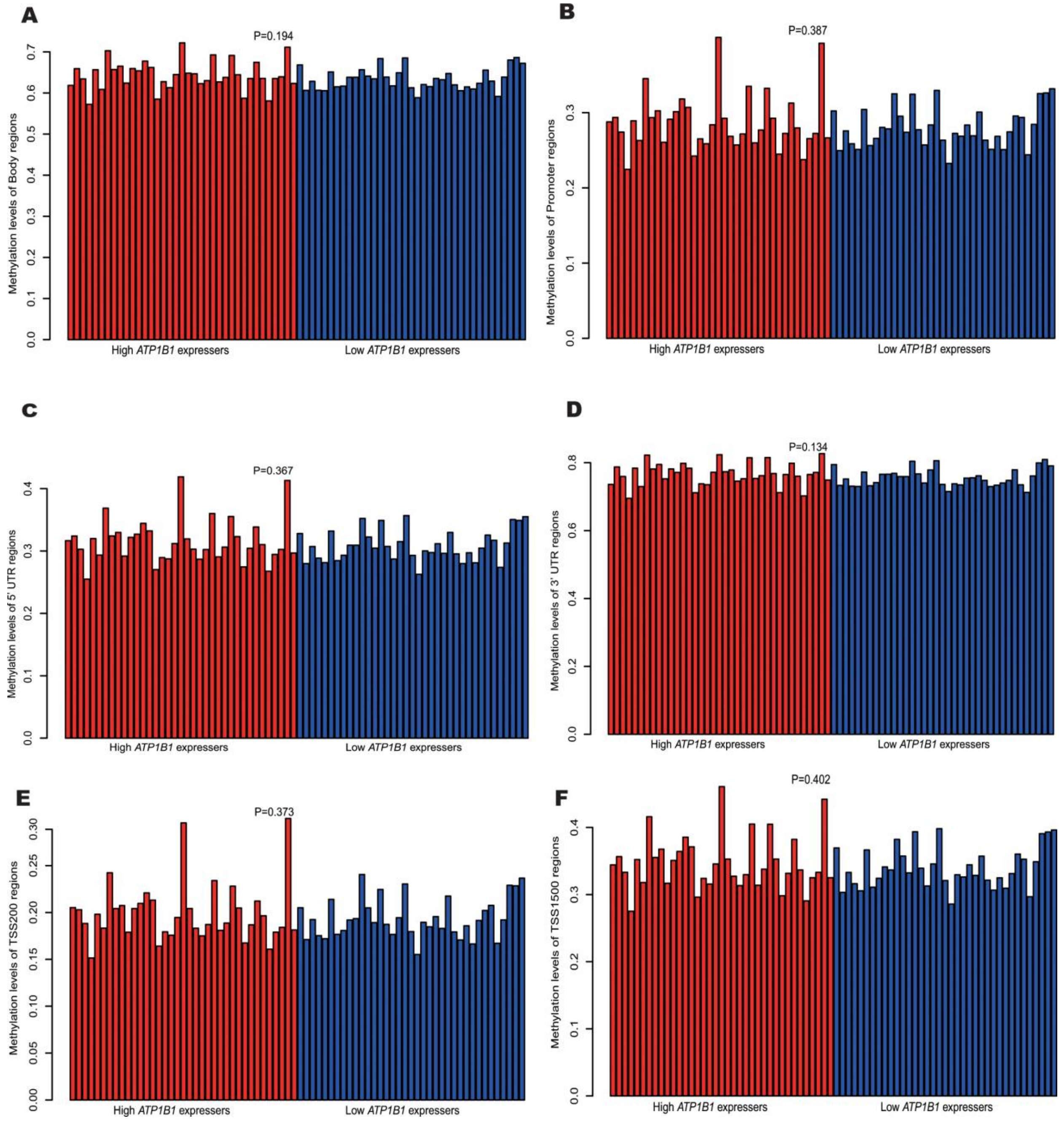

Figure S4. Methylation patterns associated with *ATP1B1* expression in different regions. (A). Body. **(B)**. Promoter**. (C)**. 5’UTR. (**D)**. 3’UTR. **(E)**. TSS200. (**F)**. TSS1500.

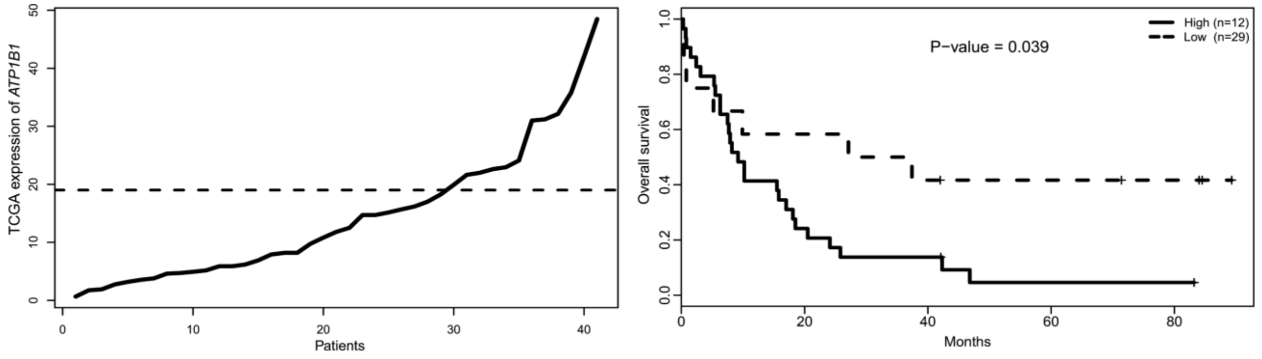

Figure S5. ***ATP1B1*** **expression and survival curve derived from TCGA high-throughput sequencing of 41 CN-AML samples. (A).** *ATP1B1* expression distribution. (**B**). Overall survival curve.

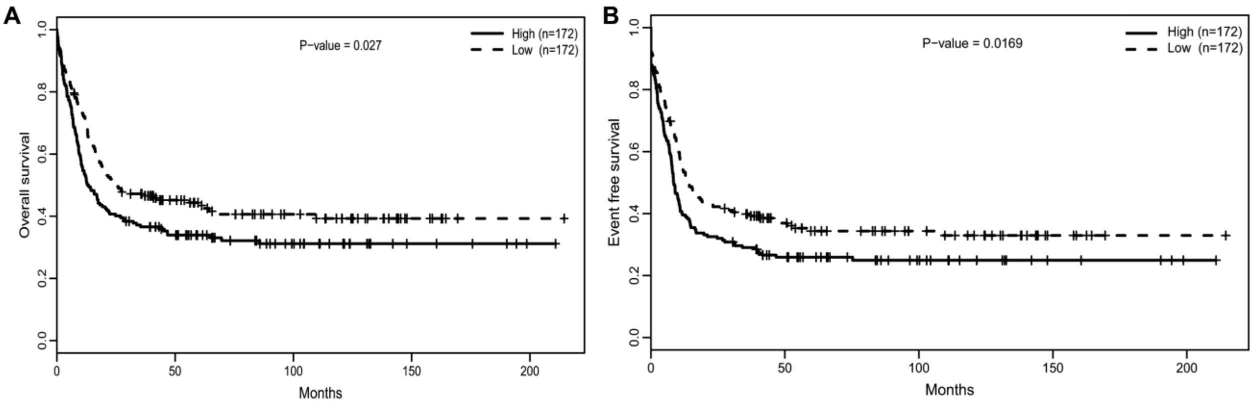

Figure S6. Overexpression of *ATP1B1* predicts adverse outcomes in a cohort of 344 AML patients. (A). OS and **(B).** EFS**.**

Table S1. Effect of *ATP1B1* expression on survival among the 157 CN-AML patients in the primary cohort.

**Table S1.** Effect of *ATP1B1* expression on survival among the 157 CN-AML patients in the primary cohort									
**Outcome**	**All patients, n=157**			**ELN Favorable category**			**ELN Intermediate-I category**		
	***ATP1B1*^{high}, n=78**	***ATP1B1*^{low}, n=79**	***P***	***ATP1B1*^{high}, n=29**	***ATP1B1*^{low}, n=30**	***P***	***ATP1B1*^{high}, n=61**	***ATP1B1*^{low}, n=61**	***P***
Mean OS (Range), m	10.87 (0.07-198.7)	37.32 (0.13-214.5)	0.023	54.51 (0.59-190.3)	67.61 (0.3-214.5)	0.19	31.08 (0.07-198.7)	47.43 (0.13-163.1)	0.028
Estimated OS at 3 y. (95% CI)	0.33 (0.24-0.46)	0.54 (0.45-0.67)	0.026	0.55 (0.4-0.77)	0.57 (0.41-0.78)	0.32	0.26 (0.17-0.4)	0.53 (0.41-0.67)	0.023
Mean (Range), m	8.15 (0.03-198.7)	21.85 (0.03-214.5)	0.04	51.09 (0.03-190.3)	51.67 (0.03-214.5)	0.5	22.57 (0.03-198.3)	39.99 (0.03-158.8)	0.057
Estimated EFS at 3 y. (95% CI)	0.27 (0.19-0.39)	0.42 (0.32-0.54)	0.012	0.51 (0.36-0.74)	0.47 (0.32-0.68)	0.4	0.18 (0.11-0.31)	0.39 (0.29-0.54)	0.004

Table S2. Clinical characteristics of the 162 CN-AML patients in the validating cohort segregated based on *ATP1B1* expression level.

**Table S2.** Clinical characteristics of the 162 CN-AML patients in the validating cohort segregated based on *ATP1B1* expression level			
**Variable**	***ATP1B1*^{high}**, n=81	***ATP1B1*^{low}**,n=81	***P***
Median age. y (range)	60 (19-83)	56 (17-79)	0.77
Median OS, d (range)	247 (3-1176)	486 (1-1176)	0.0005
FAB subtype, no.			
M0	2	3	1
M1	31	14	0.005
M2	22	23	1
M3	0	0	1
M4	20	22	0.86
M5	5	14	0.048
M6	1	5	0.21
High *ERG*, no.	54	27	<0.0001
High *BAALC*, no.	51	30	0.0016
High *LEF1*, no.	36	45	0.208
High *MN1*, no.	53	28	0.0001
High *WT1*, no.	60	21	<0.0001
High *DNMT3B*, no	54	27	<0.0001
High *TCF4*, no	55	26	<0.0001
High *ITPR2*, no	55	26	<0.0001
High *MAPKBP1*, no	49	32	0.012
High *SPARC*, no	49	32	0.012

Table S3. List of genes associated with *ATP1B1* expression.

Table S4. List of microRNAs associated with *ATP1B1* expression.
